# Tiny cystine stones in the gallbladder of a patient with cholecystolithiasis complicating acute cholecystitis: a case report

**DOI:** 10.1186/2047-783X-17-6

**Published:** 2012-03-29

**Authors:** Tie Qiao, Rui-hong Ma, Xiao-bing Luo, Yu-yang Feng, Xing-qiang Wang, Pei-ming Zheng, Zhen-liang Luo

**Affiliations:** 1 Institute of Gallbladder Disease of Panyu, Guangzhou 511470, People's Republic of China; 2 The Second People's Hospital of Panyu, Panyu, Guangzhou 511470, People's Republic of China; 3 The Second People's Hospital of Panyu, Xingye Road No. 7, Dagang Town, Panyu, Guangzhou 511470, People's Republic of China

**Keywords:** Cystine stones, Urinary calculi, Gallstones

## Abstract

Cystine stones, the main component of which is cystine, are very common urinary calculi, but are rare in the gall bladder. In animals, there has been only one report of cystine gallstones in tree shrews, and to our knowledge, this is the first report of cystine gallstones in humans.

## Background

Cholecystolithiasis, or gallbladder stone is a common and frequently encountered disease worldwide [1-4]. Gallbladder stones can be divided into four groups according to their location: intracavitary, cystic duct, intramural, and mucosal stones (small stones adhering to the mucosa) [5]. Based on their main component, gallbladder stones are divided into cholesterol stones, bile pigment stones, mixed stones, and other types [6-9] (including calcium carbonate stones, calcium phosphate stones, fatty acid stones, and cystine stones); this classification is mainly based on the infrared spectrum of the stones. Compared with other types of stones, cystine stones are rare in the gallbladder, although they are common in the urinary system. We report a patient with cystine gallstones.

## Case presentation

A 38-year-old woman with a 3-year history of cholecystolithiasis was examined at a local hospital and referred to our hospital for endoscopic gallstone removal without gallbladder excision. When she was hospitalized at our hospital, the acute right upper abdominal pain had persisted for two hours.

On physical examination, no signs of jaundice were seen in the skin or sclera. The patient's abdomen was soft, with no sign of lumps, with tenderness other than rebound tenderness in the gallbladder area. Murphy's sign was positive. Ultrasonography revealed several movable masses of 7 × 10 mm and 7 × 9 mm in size, which were strongly echogenic, with acoustic shadowing in the body of the gallbladder; an immovable strongly echogenic mass of 7 × 10 mm with acoustic shadowing in the neck of gallbladder and cystic duct; and poor sound penetration and a dense, low-light spot in the dark space of the bile. There was no thickening of the gallbladder wall. The gallbladder emptying index was 30%. No obvious abnormalities were seen in the liver, spleen or pancreas.

The patient was diagnosed as having cholecystolithiasis complicating acute cholecystitis and incomplete biliary-tract obstruction. The patient had been given anti-inflammatory treatment after the examination for 2 weeks and the symptoms of acute cholecystitis had disappeared before the operation was schedule, and the patient was strongly in favor of preserving the gallbladder.

**Figure 1:**
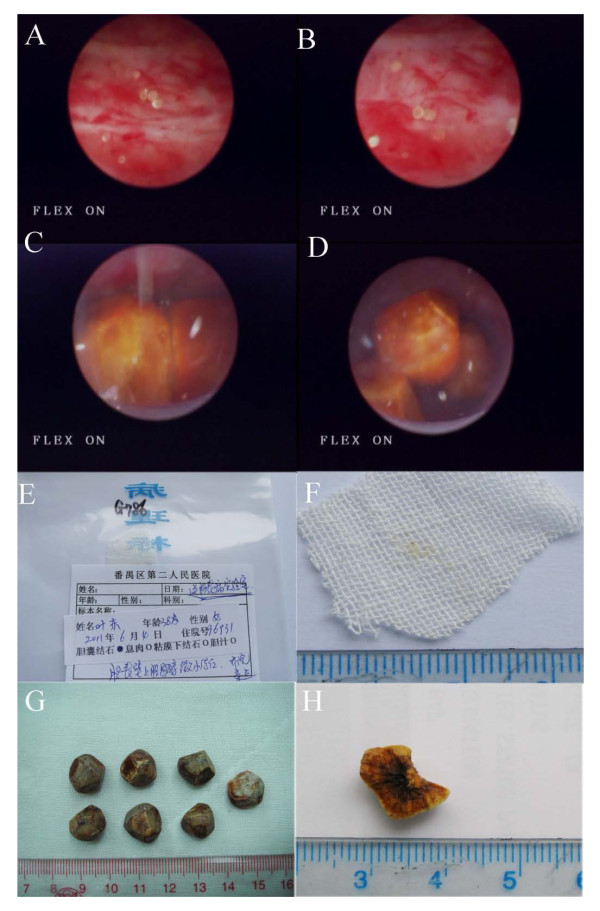
**Endoscopic and gross appearance of the stones. (A,B) **Small stones and purulent bile and **(C,D) **large stones and purulent bile under endoscopy. **(E-H) **Appearance of **(E,F) **small stones and **(G,H) **large stones with a radial, layered arrangement in profile.

**Figure 2:**
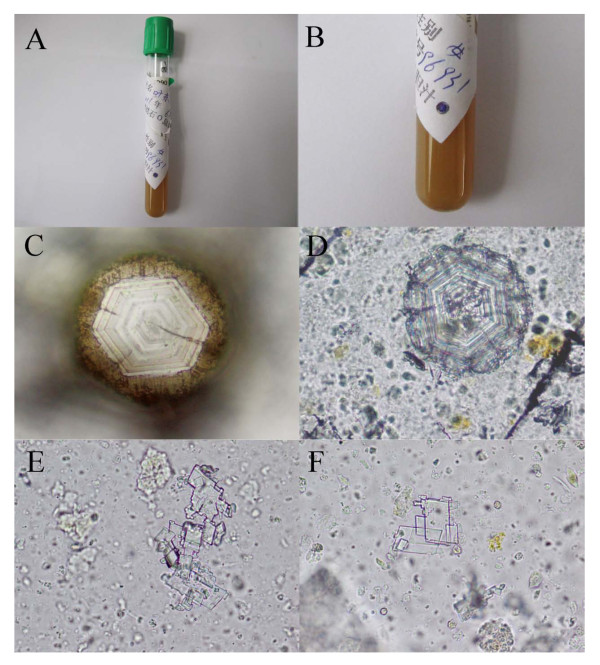
**Appearance and microscopic examination of the bile. (A,B) **Appearance of the bile. **(C,D) **Cystine and **(E,F) **cholesterol crystals under light microscopy.

Consequently, after approval by the medical ethics committee and provision of informed consent by the patient, the gallbladder was laparoscopically isolated and transected at the bottom (< 6 mm) under general anesthesia. First, the bile was drained with a sterile ventricular drainage tube to a sterile injector, and transferred to sterile tubes. Next, the gallbladder was explored with a three-channeled cholecystoscope (CHiAO; Chinese national patent number: ZL200810026985.X HAWK, China [10]), and the stones were collected with a stone extractor. We found only mild congestion in the gallbladder mucosa, which indicated only slight inflammation of gallbladder. Using endoscopy, we found many small, semitransparent stones (< 1 mm) adhered to the gallbladder wall (Figure 1A, B). These stones were removed with an endoscopic attachment (CHiAO absorbing box; Chinese national patent number: ZL 201110167069.X) designed to remove sludge-like gallstones combined with seven types of manipulation (pushing, squeezing, pressuring, tearing, bracing, flushing, and sucking) [5], while several large stones (> 5 mm) were removed with a stone extractor (Figure 1C, D). The small stones were yellowish and semitransparent, and the large stones were polyhedron or globular in shape, and had a radial, layered arrangement in profile (Figure 1E-H). The bile was yellowish, opaque, turbid, and very viscous. After centrifugation at 1,450 *g *for 10 min, the bile supernatant was transferred to a clean tube, and about 0.5 mL of sediment was kept. The bile sediment was smeared onto labeled slides and viewed under a system microscope (BX51; Olympus, Tokyo, Japan). Colorless hexagonal plate crystals with high refractivity and limpid edges were seen, which were judged to be cystine crystals based on their morphology. Meanwhile, colorless, transparent crystals (in the shape of rectangles with missing corners or squares with missing corners, or glass flakes) were seen, which were judged to be cholesterol crystals from their morphology (Figure 2).

**Figure 3:**
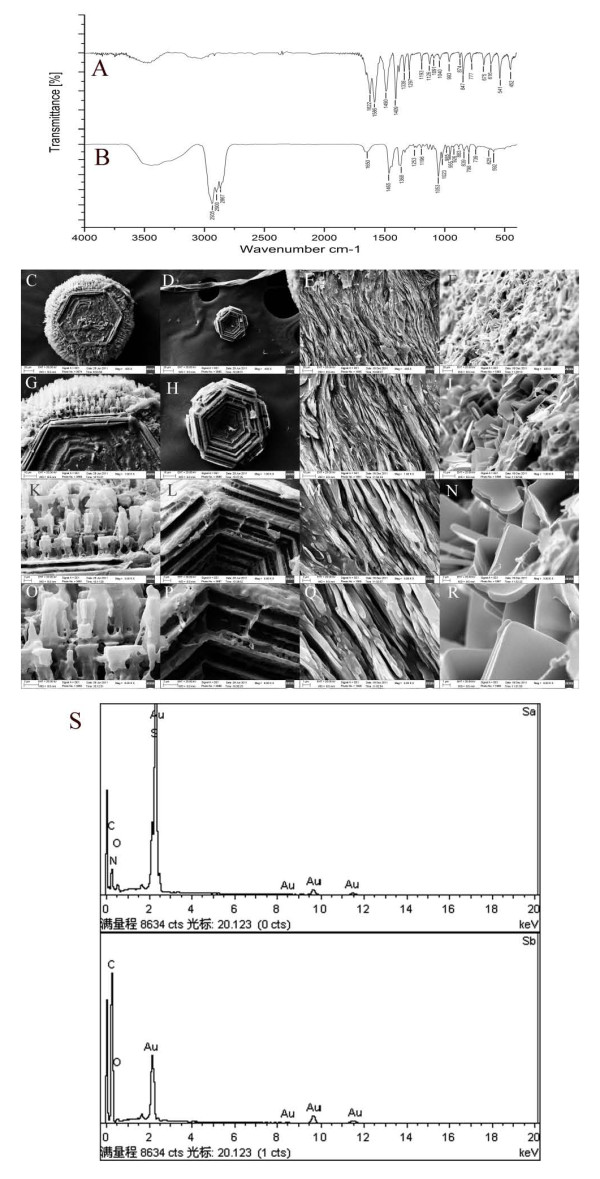
**Results of analysis with spectrogram and electron microscope. (A,B) **FTIR spectrogram of **(A) **small stones, showing that the main component was cystine, and **(B) **of the large stones, showing that the main component was cholesterol. **(C-R) **Scanning electron microscopy of the small cystine and large cholesterol stones. The small cystine stones displayed **(C,D) **hexagon crystals of unequal size, some with prominences on the edge (original magnification ×400) and **(G,H,K,L) **hexagon crystals stacked and arranged tightly. Original magnification (G,H) ×1,000; (K,L) ×3,000. **(O,P) **A view of one corner of a hexagon crystal shows that the end of the prominence is lamellar (original magnification ×6,000). The large cholesterol stones showed **(E,F) **amellar cholesterol crystals (original magnification ×400) and **(I,J,M,N) **stacked lamellar cholesterol crystals, Original magnification (I,J) ×1,000; (M,N) ×3,000. **(Q,R) **The cholesterol crystals had a smooth, glossy surface, dull borders, and a pyknomorphous texture (original magnification×6,000). **(S) **X-ray energy spectrogram Sa, spectrogram of the small stones (the elemental composition was carbon, oxygen, nitrogen, and sulfur) Sb, spectrogram of the large stones (the elemental composition was carbon and oxygen).

The gallbladder stones were analyzed with a Fourier transform infrared spectrometer (TENSOR27; Bruker Optics GmBH, Ettlingen, Germany) in the frequency range of 400 to 4,000 per cm at 4 per cm resolution. The results indicated that the small stones were cystine stones and that the large stones were cholesterol stones; (Figure 3A, B).

Next, some of the small stones and a piece of a large stone were fixed onto the sample table and dried at 60°C overnight, then sputter-coated with gold (ETD-2000, Beijing Elaborate Technology Development Ltd., China) and observed under a scanning electron microscope (EVO LS10; Carl Zeiss, Cambridge, England). The samples were photographed and analyzed with an energy spectrometer (X-Max; Oxford Instruments plc, Oxford, UK). Under scanning electron microscopy, the small stones were found to be composed of hexagonal cystine crystals (30-270 μm), some with prominences on their edges. The energy spectrum indicated that the elemental composition was carbon, oxygen, nitrogen, and sulfur, along with gold from the coating. The large stones were composed of lamellar cholesterol crystals with a thickness of about 1 μm. The energy spectrum indicated that the elemental composition was carbon and oxygen, with gold from the coating (Figure 3C-S).

## Conclusions

The spectrum of the small stones indicated that the main component was cystine, and the energy spectrum revealed that the main elements were carbon, oxygen, nitrogen, and sulfur, confirming that these stones were composed of cystine. The spectrum of the large stones indicated that the main component was cholesterol, and the energy spectrum confirmed this, showing that the main elements were carbon and oxygen.

There are no reports of cystine gallstones in any species, with the exception of one report describing sick tree shrews [11], and in that case, the pathogenesis was not clear. There have been no reports in humans. Urinary cystine stones are mainly induced by cystinuria, an inborn error of metabolism [12-15]. Supersaturation of cystine in the urine leads to the precipitation of cystine crystals, and subsequent formation of cystine stones. We found cystine crystals in our patient's bile sediment, which indicated that the pathogenesis of cystine stones in the gallbladder may be similar to that in the urinary system. However, the patient did not have urinary calculi. It is possible that the presence of stones in the cystic duct led to the incomplete obstruction of the biliary tract and a change in bile metabolism, resulting in the formation of cystine stones.

The mechanism of the formation of cholesterol gallstones involves three main components: supersaturation of cholesterol, gallbladder hypomotility, and kinetic factors [16-26]. Cholesterol is slightly soluble in aqueous media, but is made soluble in bile by forming mixed micelles with bile salts and lecithin. An increase in lecithin or a decrease in bile acid will lead to supersaturation of cholesterol, which produces crystals and precipitates around the core, with spiral growth from the center outwards, and a radial, layered, cord-like arrangement of cholesterol crystals, culminating in stone formation. Patients with incomplete gallbladder emptying were found to have increased total lipid concentrations and some proteins that promote stone formation [20]. The main mechanism for the formation of cholesterol stones, which involves cholesterol supersaturation, gallbladder hypomotility, and kinetic factors, may also lead to the formation of other types of gallstones. Our patient had a normal blood lipid level and a gallbladder emptying index of 30%, indicating poor gallbladder function. These factors may be involved in the formation of both cholesterol and cystine stones.

In conclusion, we report a case of cystine gallstones, a rare type of gallstone, combined with cholesterol stones, in a patient with cholecystolithiasis complicating acute cholecystitis and incomplete obstruction of the biliary duct. The tiny cystine gallstones were adhered to the gallbladder wall and therefore difficult to locate. Surgeons should be aware of the possibility of such stones and ensure that they are removed to reduce the possibility of recurrence. The patient was followed up for 6 months with no abnormal occurrence or recurrence, indicating that the gallstone removal without gallbladder excision has been effective, but she will need to have a longer period of follow-up.

The pathogenesis of cystine stone formation in the gallbladder is not clear, and requires further research. However, this case report provides new insight into the pathogenesis of gallstones. The present research indicates that the composition and genesis of gallstones is more complicated than previously thought, and requires further research and exploration.

## Consent

Written informed consent was obtained from the patient for publication of this Case report and any accompanying images. A copy of the written consent is available for review by the Editor-in-Chief of this journal.

## Abbreviations

CS: Cystine stones; UC: Urinary calculi; GS: Gallstones; CS: Cholesterol stones; BPG: Bile pigment stones; MS: Mixed stones; CCS: Calcium carbonate stones; CPS: Calcium phosphate stones; FAS: Fatty acid stones; FTIR: Fourier transform infrared spectroscopy; SEM: Scanning electron microscope; XRES: X-ray energy spectrometer.

## Competing interests

The authors declare that they have no competing interests.

## Authors' contributions

TQ, study concept and design; RM, acquisition of data; analysis and interpretation of data; drafting of the manuscript; XL, critical revision of the manuscript for important intellectual content; YF, material support; XW, material support; PZ, technical, or material support; ZL, technical, or material support. All authors read and approved the final manuscript.

## Authors' information

Tie Qiao, bachelor's degree, Superintendent of the institution, surgeon; Rui-hong Ma, master's degree, researcher; Xiao-bing Luo, master's degree, researcher; Yu-yang Feng, bachelor's degree, surgeon; Xing-qiang Wang, bachelor's degree, surgeon; Pei-ming Zheng, master's degree, researcher; Zhen-liang Luo, doctor's degree, researcher.
